# The Neurovascular Unit in Dementia: An Opinion on Current Research and Future Directions

**DOI:** 10.3389/fnagi.2021.721937

**Published:** 2021-07-28

**Authors:** Lucy Beishon, Ronney B. Panerai

**Affiliations:** ^1^Department of Cardiovascular Sciences, University of Leicester, Leicester, United Kingdom; ^2^National Institute for Health Research Leicester Biomedical Research Centre, British Heart Foundation Cardiovascular Research Centre, Glenfield Hospital, Leicester, United Kingdom

**Keywords:** Alzheimer's disease, mild cognitive impairment, cerebrovascular disease, cognitive dysfunction, neurovascular coupling

## Introduction

Dementia is a burgeoning public health crisis, with 50 million people currently affected worldwide (Prince et al., 2015). As the population ages, this figure is set to rise dramatically by 40% over the next 12 years (Prince et al., 2015). Dementia is an umbrella term for several disorders which result in the progressive loss of memory or other cognitive functions (Scott and Barrett, 2007). It remains an incurable disease, and current therapeutics have limited efficacy at slowing disease progression for one third of patients (Rockwood et al., 2008). Of the dementia sub-types, Alzheimer's disease (AD) remains the most prevalent, accounting for ~60–70% cases (Alzheimer's-Society, 2016). Vascular dementia (VaD) is the second most common form and is responsible for ~20% of cases, with a further 10% being a combination of these two diseases (Alzheimer's-Society, 2016). However, in practise these distinctions are somewhat arbitrary given the significant overlap in altered vascular structure and function in both of these major sub-types (Kalaria and Ballard, 1999). At least 30% of patients with AD have evidence of cerebrovascular disease on post-mortem examination, and almost all have evidence of cerebral amyloid angiopathy, microvascular degeneration, and white matter lesions (Kalaria and Ballard, 1999). Similarly, one-third of patients with VaD exhibit pathology consistent with AD (e.g., hippocampal or temporal lobe atrophy) (Kalaria and Ballard, 1999). Longitudinal studies have demonstrated that vascular risk factors (e.g., hypertension), significantly increase the risk of both AD and VaD (Rius-Pérez et al., 2018). In genetically at-risk individuals positive for apolipoprotein E4 (APOE4), atherosclerosis can increase the risk of AD by three-fold (Hoffmann et al., 2010). This article provides an opinion on the current evidence on the role of the neurovascular unit in dementia, for further information, several recent reviews are available on this topic (Nelson et al., 2016; Kisler et al., 2017).

## Amyloid Cascade Hypothesis

A number of mechanistic models have been proposed to understand the pathological basis of AD. The amyloid cascade hypothesis gained increasing traction over the last few decades, having dominated the research sphere (Morris et al., 2014). Amyloid-based biomarkers have been incorporated into a number of diagnostic guidelines (Jack et al., 2018), and the histopathological (gold standard) diagnosis of AD includes the presence of amyloid plaque and neurofibrillatory tangles (Deture and Dickson, 2019). However, despite decades of research into this hypothesis, and several large trials of amyloid based drugs, none have demonstrated efficacy warranting their widespread use in clinical practise (Morris et al., 2014). Only tramiprosate, a selective anti-oligomer agent, has demonstrated potential benefit for a sub-group of APOE4 positive individuals with early AD and is currently under investigation in a phase three trial (Tolar et al., 2020). These findings have raised several questions around the amyloid cascade hypothesis. Firstly, the lack of efficacy for amyloid-based targets may suggest amyloid is a by-product rather than causative agent of the disease process. This is supported by the finding that amyloid deposition commonly occurs in cognitively healthy older adults, and plaque burden does not correlate well with the level of cognitive deficit (Morris et al., 2014). In contrast, synaptic loss, microglial activation, neurofibrillatory tangles, and cerebral blood flow correlate better with disease severity in AD (Rius-Pérez et al., 2018). Secondly, the potential efficacy in a sub-group of early AD (Tolar et al., 2020) suggests that amyloid is a late occurrence in the disease process, at which stage irrevocable damage and cognitive decline has ensued. Furthermore, only patients with a strong genetic risk may benefit from these therapies (Tolar et al., 2020), limiting the wider applicability of these drugs. These unanswered questions have thus stimulated the search for earlier potential therapeutic targets, particularly those which are identifiable at earlier stages, preceding the development of cognitive decline and amyloid deposition.

## Vascular Cascade Hypothesis

The vascular cascade hypothesis postulates that early disruption of vascular mechanisms as a result of sustained vascular risk factors and poor lifestyle habits, results in a state of chronic hypoperfusion (Rius-Pérez et al., 2018). This leads to the development of blood brain barrier breakdown, tau hyperphosphorylation, and amyloid deposition (Nelson et al., 2016). The blood brain barrier is essential to maintain a tightly controlled environment, and contributes to the clearance of amyloid-beta (Rius-Pérez et al., 2018). BBB dysfunction has been demonstrated to occur in the hippocampus with normal ageing (Montagne et al., 2015), early AD (Nation et al., 2019), and in APOE4 positive individuals (Montagne et al., 2020). As a result, amyloid deposition damages the cerebrovasculature, both structurally and functionally, therefore worsening hypoperfusion in a cyclical fashion (Nelson et al., 2016). These findings led to the development of the two-hit hypothesis, where the vascular insult represents the first “hit” to the system, followed by the amyloid or second “hit,” with the two processes subsequently interacting in a dynamic manner to worsen hypoperfusion, increase tau hyperphosphorylation, and promote amyloid deposition (Nelson et al., 2016). Importantly, the vascular hit is thought to occur earlier in the disease process (Hays et al., 2016). This notion is supported by longitudinal studies of ageing demonstrating that alterations in cerebral haemodynamics are detectable in cognitively intact older adults, and are predictive of future dementia risk (Wolters et al., 2017).

## The Neurovascular Unit in Dementia

The neurovascular unit is formed by the neurone and its supporting cells (astrocytes, endothelial cells, pericytes, and smooth muscle cells) (Iadecola, 2017). They are closely related both structurally and functionally to ensure the tight coupling of neuronal activity and cerebral blood flow, termed neurovascular coupling (NVC) (Iadecola, 2017). This is achieved through feedforward and feedback mechanisms as a result of the release of active metabolites and chemical mediators (Hosford and Gourine, 2019). De-coupling of these processes has been shown to occur in animal models of AD (Girouard and Iadecola, 2006). Human studies have demonstrated conflicting findings of both increased (Corriveau-Lecavalier et al., 2019), and decreased (Beishon et al., 2018) vascular responses to cognitive stimulation. These opposing findings may reflect compensatory mechanisms occurring early in the disease process, vs. the failure of these mechanisms at later stages (Merlo et al., 2019).

Therefore, deficiencies occurring in one or more components of the NVU threaten this tightly coordinated system. Inadequate matching of perfusion to neuronal activity will fail to clear the active metabolites generated by a resource intensive process, the accumulation of which can result in neurotoxicity (Girouard and Iadecola, 2006). Furthermore, inadequate perfusion will limit the provision of oxygen and glucose, essential for optimal neuronal function and cell signalling, thus limiting the capacity for cognitive function (Girouard and Iadecola, 2006).

## The NVU as a Biomarker and Therapeutic Target in Dementia

As a result of these findings, increasing interest in the NVU as both a biomarker and therapeutic target has emerged. A number of neuroimaging based methods have been used to detect abnormalities in cerebral haemodynamics occurring in healthy, mildly impaired, or established dementia (Hays et al., 2016). A number of neuroimaging based biomarkers have been investigated, and can be broadly divided into portable and non-portable based techniques. Portable techniques have the advantage of providing a simple, bedside measurement with excellent temporal resolution and continuous monitoring of haemodynamic measures (Panerai, 2009; Balardin et al., 2017). Studies measuring metabolic changes, as a proxy for perfusion, have demonstrated good sensitivity and specificity to differentiate stable and progressive forms of mild cognitive impairment (MCI) (Henderson, 2012; Marcus et al., 2014). However, many of these techniques remain confined to the research domain, and are only recommended where the diagnosis remains uncertain (National Institute for Health Care Excellence, 2018).

In terms of vascular targets, the majority of research has focussed on currently available treatments to modify vascular risk, such as antihypertensive drugs (Bhat, 2015). Given the extensive evidence supporting a role for vascular mechanisms in the development of AD, modification of vascular risk is an attractive and amenable target. However, to gain benefit, these factors are likely to need controlling in mid-life given that these risks translate into cognitive decline over a sustained and longer period (Livingston et al., 2020). Furthermore, the role for vascular risk, and particularly blood pressure reduction, remains uncertain for people with established dementia (Harrison et al., 2016). A recent Cochrane review found limited evidence to support antihypertensive withdrawal in dementia, and may result in increased cardiovascular events (Jongstra et al., 2016). Data from observational studies suggest cerebral autoregulation remains intact in MCI and dementia (De Heus et al., 2018), and a recent study demonstrated improved hippocampal CBF in patients with dementia treated with nilvadipine (Jong et al., 2019). The RADAR trial is currently ongoing, and will investigate the effects of losartan in mild to moderate AD on brain atrophy, white matter hyper intensities, and cerebral blood flow (Kehoe et al., 2018). Recently, interest has been gaining momentum on the effects of lifestyle interventions (exercises, diet, cognitive intervention) on cerebrovascular function, and whether multi-modal interventions can promote vascular brain health. In two recent systematic reviews (Beishon et al., 2020), cognitive training has been demonstrated to alter brain volumes and functional connectivity in MCI and dementia, but few studies have specifically investigated their effects on vascular mechanisms. Finally, novel therapeutic targets have been proposed around the various components of the NVU (Zlokovic, 2011). Vasculoprotective agents that target blood brain barrier function (e.g., activated protein C) and promote integrity are promising (Zlokovic, 2011). Similarly, mediators that promote angiogenesis (vascular endothelial growth factor) or improve amyloid-beta clearance (insulin like growth factor) may also be beneficial (Zlokovic, 2011).

## Discussion

In summary, vascular mechanisms play a key role in development and progression of cognitive dysfunction. Importantly, disruption to vascular physiology occurs early in the disease process, providing a potential target to prevent or delay the onset of dementia. Despite this breadth of evidence demonstrating both structural and functional damage to the cerebrovascular system in early dementia, few vascular targets have been the subject of large-scale randomised controlled trials. Disappointingly, in a recent review, few trials employed agents or targets of vascular dysfunction (Huang et al., 2020). This suggests more work is needed in both animal models to identify potential targets, and in patients to take these targets to clinical trials. Importantly, the identification of new targets has been hampered by a lack of translation between animal models and clinical trials (Cavanaugh et al., 2014). Current transgenic animal models of AD most closely represent inherited forms of AD, which are not the dominant phenotype seen in clinical practise (Cavanaugh et al., 2014). These models will have a bias towards amyloid-based pathology, and may not reflect the alterations to vascular structure and function seen in humans, particularly with late-onset AD. Furthermore, the amyloid pathology in animal models does not correlate well with that seen in humans, suggesting there are key differences in the pathological basis of AD development between species (Cavanaugh et al., 2014). BBB dysfunction has been demonstrated in animal models of AD (Montagne et al., 2017), but amongst genetic-based models which may be pathologically distinct from late onset AD seen clinically. In addition to drug-based targets, research is urgently needed to clarify the role of lifestyle interventions on cerebrovascular disease in dementia risk reduction and treatment. Lifestyle interventions are resource intensive, and can be physically and mentally demanding for people with dementia to undertake. The Finnish Geriatric Intervention Study to Prevent Cognitive Impairment and Disability (FINGER) randomised at-risk older adults to an intensive programme of diet, exercise, cognitive training, and vascular risk monitoring, lasting 2 years (Ngandu et al., 2015). The trial found small benefits to cognitive function in the intervention group, with a drop-out rate of ~12% (Ngandu et al., 2015). Given that benefits to cognitive function tend to be small, and the long trajectory to cognitive decline, cerebrovascular biomarkers as a surrogate for clinical outcome measures could be beneficial in reducing the durations required for clinical trials to demonstrate effectiveness. However, limited information is available on the effects of such multi-modal interventions on cerebrovascular function, and their relationship to longer term clinical outcomes. Future trials of lifestyle interventions would benefit from the addition of cerebrovascular outcomes to understand the effects on vascular structure and function, which could contribute to the identification of novel therapeutic targets.

## Author Contributions

LB and RP jointly drafted the manuscript. All authors contributed to the article and approved the submitted version.

## Conflict of Interest

The authors declare that the research was conducted in the absence of any commercial or financial relationships that could be construed as a potential conflict of interest.

## Publisher's Note

All claims expressed in this article are solely those of the authors and do not necessarily represent those of their affiliated organizations, or those of the publisher, the editors and the reviewers. Any product that may be evaluated in this article, or claim that may be made by its manufacturer, is not guaranteed or endorsed by the publisher.
